# Detection of Human Herpes Virus 8 in Kaposi’s sarcoma tissues at the University Teaching Hospital, Lusaka, Zambia

**DOI:** 10.11604/pamj.2017.27.137.11845

**Published:** 2017-06-22

**Authors:** Rabecca Tembo, Trevor Kaile, Doris Kafita, Chrispin Chisanga, Annie Kalonda, Ephraim Zulu, Mulemba Samutela, Pascal Polepole, Geoffrey Kwenda

**Affiliations:** 1 Department of Pathology and Microbiology, School of Medicine, University of Zambia, Lusaka, Zambia; 2 Department of Biomedical Sciences, School of Health Sciences, University of Zambia, Lusaka, Zambia

**Keywords:** Human Herpes Virus 8, Kaposi´s sarcoma, histological types

## Abstract

**Introduction:**

Human herpes virus-8, a γ2-herpes virus, is the aetiological agent of Kaposi sarcoma. Recently, Kaposi's sarcoma cases have increased in Zambia. However, the diagnosis of this disease is based on morphological appearance of affected tissues using histological techniques, and the association with its causative agent, Human Herpes virus 8 is not sought. This means poor prognosis for affected patients since the causative agent is not targeted during diagnosis and KS lesions may be mistaken for other reactive and neoplastic vascular proliferations when only histological techniques are used. Therefore, this study was aimed at providing evidence of Human Herpes virus 8 infection in Kaposi's sarcoma tissues at the University Teaching Hospital in Lusaka, Zambia.

**Methods:**

One hundred and twenty suspected Kaposi's sarcoma archival formalin-fixed paraffin-wax embedded tissues stored from January 2013 to December 2014 in the Histopathology Laboratory at the University Teaching Hospital, Lusaka, Zambia were analysed using histology and Polymerase Chain Reaction targeting the ORF26 gene of Human Herpes virus 8.

**Results:**

The predominant histological type of Kaposi's sarcoma detected was the Nodular type (60.7%) followed by the plaque type (22.6%) and patch type (16.7%). The nodular lesion was identified mostly in males (40.5%, 34/84) than females (20.2%, 17/84) (p=0.041). Human Herpes virus 8 DNA was detected in 53.6% (45/84) and mostly in the nodular KS lesions (60%, 27/84) (p=0.035).

**Conclusion:**

The findings in this study show that the Human Herpes virus-8 is detectable in Kaposi's sarcoma tissues, and, as previously reported in other settings, is closely associated with Kaposi's sarcoma. The study has provided important baseline data for use in the diagnosis of this disease and the identification of the virus in the tissues will aid in targeted therapy.

## Introduction

Human herpes virus-8 (HHV-8), a γ2-herpes virus, also known as Kaposi sarcoma-associated herpesvirus (KSHV), is the aetiological agent of Kaposi sarcoma(KS) [1–3] and is also aetiologically linked to two other lymphoproliferative disorders, Primary Effusion Lymphoma (PEL) and Multicentric Castleman's disease (MCD) [2, 4, 5], which have rarely been reported in Africa [6]. KS is a mesenchymal tumour involving blood and lymphatic vessels and was first described in Eastern Europe in the late 19th century and classically considered as an indolent disease of elderly men [7–9]. This malignancy is predominantly seen in people with acquired immunodeficiencies, including acquired immunodeficiency syndrome (AIDS) and iatrogenic immunosuppression in the setting of organ transplantation, but can also develop in the elderly [1, 9]. KS is most frequent in regions with high HHV-8 seroprevalence, such as sub-Saharan Africa and some Mediterranean countries [10, 11]. The global seroprevalence of HHV-8 is uneven. It is low in the United States and Western Europe, moderate in the Mediterranean and as high as 80% in sub-Saharan Africa [12–14].

KS is now the most frequently reported malignant skin tumour in some areas of Africa and was endemic in Africa even before the advent of Human Immunodeficiency Virus (HIV) infection [15, 16]. It remains the most frequent HIV-associated malignancy and major scourge in sub-Saharan Africa, especially in the so-called “KS” belt, which covers Kenya, Uganda, Tanzania, Zambia, Zimbabwe and South Africa [14, 17–19]. Detection of HHV-8 provides important epidemiological data which can be used to determine the occurrence, prevalence and spread of KS [1]. Therefore, the objective of this study was to detect HHV-8 in kaposi's sarcoma tissues at the University Teaching Hospital, Lusaka, Zambia.

## Methods

**Specimens:** The study was a laboratory-based retrospective study on 120 archival formalin-fixed paraffin-embedded (FFPE) KS tissues. It study was conducted at the University Teaching Hospital (UTH) in the Department of Pathology and Microbiology, Histopathology Laboratory in Lusaka. The UTH is a tertiary referral and teaching hospital with a bed capacity of about 1,664. The hospital has about 11 departments namely: Obstetrics and Gynaecology, Paediatrics, Surgery, Community medicine, Pathology, Radiology, Physiotherapy, Pharmacy and Blood bank. It is the biggest referral hospital and the centre for all histopathology diagnostic work in Zambia.

**Histological examination:** Sections of tissue were cut at 6μm on a Shandon Finesse 325 microtome (Thermo Scientific-Shandon, California, USA). The tissue sections were then stained using Haematoxylin and Eosin staining and examined by a qualified pathologist. The KS lesions were grouped into patch, plaque and nodular histological stages.

**DNA extraction:** Tissue sections were cut as described above. Up to 30mg of tissue sections was placed in a sterile 1.5ml microfuge tube. DNA was extracted using the EZNA Tissue DNA Extraction Kit (Omega Bio-Tek Inc, Norcross, Georgia, USA) for paraffin-embedded tissue according to the manufacturer´s protocol. The DNA was eluted in 50μl volumes, and then stored at -20°C until required.

**PCR amplification and detection of HHV-8 ORF26 gene:** DNA extracted was used for the detection of the HHV-8 sequences. Nested PCR was performed using two sets of primers, KS-1(5'-AGCCGAAAGATTCCACCAT-3') and KS-2 (5'-TCCGTGTTGTCTACGTCCAG-5'), KS-4(5'-CGAATCCAACGGATTTGACCTC-3') and KS-5(5'-CCCATAAATGACACATTGGTGGTA-3') amplifying the ORF26 gene of the HHV-8 genome. The 2 sets of primers were used for screening the DNA for HHV-8. The first round PCR reactions were performed in a final volume of 25μl of which 2μl was genomic DNA, 1X PCR buffer, 3.5 mM MgCl2, 0.2mM of deoxynucleoside triphosphates, and 0.2 U of Invitrogen Taq DNA polymerase (Thermo scientific co Ltd, USA ), 2.5μl of each primer on a Gene amplification 2700 Thermocycler (Applied Biosystems, CA, USA). Primers were combined at a final concentration of 0.2μM. For the second-round PCR, 2μl of the first-round PCR products was used as the template DNA. The cycling procedure for the KS-1, KS-2 primer pair was 94°C for 5 minutes, then 35 cycles of 94°C for 30 seconds, 58°C for 30 seconds, 72°C for 30 seconds and 1 final extension cycle of 7 minutes at 72°C. For the KS-4 and KS-5 primers, the amplification conditions were similar except the annealing temperature was at 53°C. The PCR products were analysed by electrophoresis on a 1.5% agarose gel containing ethidium bromide (10μg/ml in distilled water) and were visualized under ultraviolet light alongside a 50bp DNA ladder using the Biotop SC-645 Gel Documentation system (Biotech Co. Ltd, Shanghai, China). A known KS case was used as a positive control and a negative control containing nuclease-free without DNA was always included. The expected product size was 233bp.

As an internal control, the extracted DNA was subjected to PCR using primers for human β-actin (Forward 5'-GCC ATG TAC GTT GCT ATC C-3' and Reverse 5'-CCG CGC TCG GTG AGG-3') with the following conditions: initial denaturation of 94°C for 1 minute, 35 cycles of 95°C for 30 seconds, 62°C for 1minute, 72°C for 1 minute and 1 final extension cycle of 10minutes at 72°C. The PCR products were also analysed by electrophoresis on a 1.5% agarose gel containing ethidium bromide (10μg/ml) and visualised under ultraviolet light alongside the 50bp DNA ladder. The expected product size was 200bp.

**Statistical analysis:** Statistical analysis was performed using GraphPad Prism Software Version 6 (San Diego, California, USA). Logistic regression was used to determine associations among HHV-8, histological types of KS, gender and age. Chi-square and Fisher's exact test were used to determine associations among histological types of KS, gender and age. A p-value of less than 0.05 was chosen to indicate statistical significance.

**Ethics considerations:** This study was laboratory-based with no direct contact with patients. Permission to use the archived histopathology tissues was sought from the Head of the Department of Pathology and Microbiology at the University Teaching Hospital. Ethics approval for the research was sought from the University of Zambia Biomedical and Research Ethics Committee (UNZABREC) (Ethics clearance number: 002-04-14/B).

## Results

**Figure 1:**
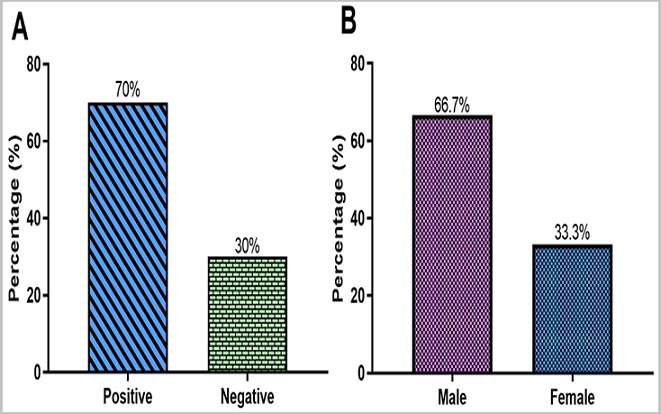
A) determination of KS cases; B) KS cases based on gender

**Figure 2:**
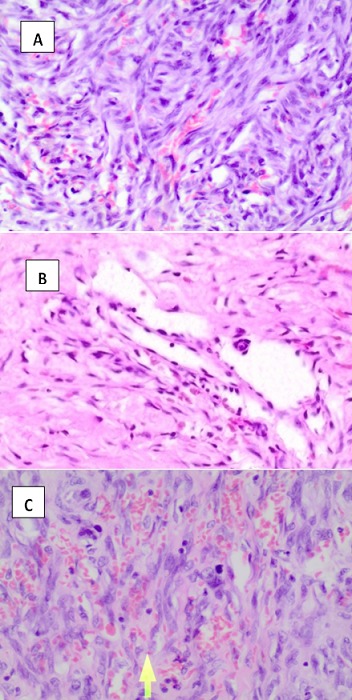
Histopathological features of KS lesions (H and E staining); A) shows the patch stage: dissection of collagen bundles by slit-like vascular channels lined by a monolayer of relatively flattened endothelial cells, variable degree of erythrocyte extravasation; B) plaque stage: proliferation of spindle cells and extravasation of erythrocytes in slit-like vascular channels; C) nodular stage of KS with formation of well-defined nodules, more spindle cell proliferation and erythrocyte extravasation, disappearance of all normal features of the skin (Adapted from the University Teaching Hospital- Histopathology Laboratory)

**Figure 3:**
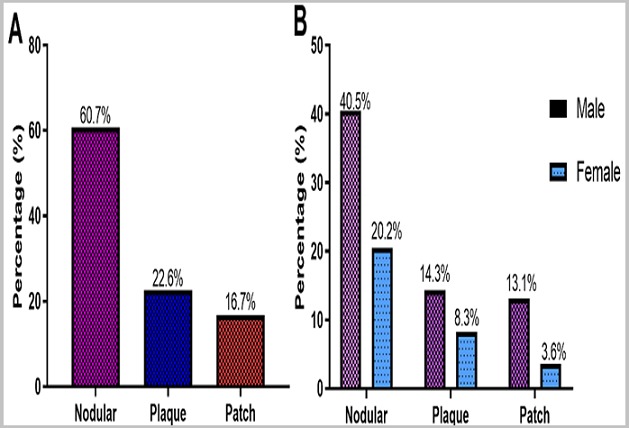
A) histological Types of KS identified; B) identification of Histological Types of KS based on gender

**Figure 4:**
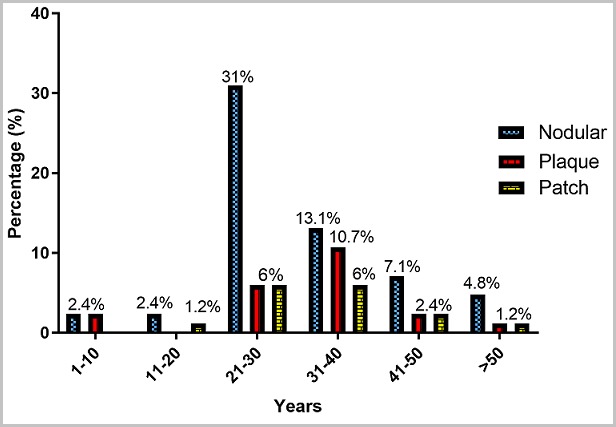
Identification of histological types of KS based on age

**Histology:** Out of the 120 suspected FFPE KS tissues analysed, 70% (84/120) were confirmed KS cases in mostly males (64.4%, 56/84) (Figure 1 A) than females (33.3%, 28/84) (Figure 1 B). The KS lesions were grouped into three histological types: Patch (early vascular lesion), plaque (intermediate lesion) and nodular (advanced lesion) based on the features seen under the microscope as shown in Figure 2. The histological type distribution was as follows: Nodular (60.7%, 51/84), Patch (22.6%, 19/84), and Plaque (16.7%, 14/84) (Figure 3 A). The nodular type was the commonly diagnosed histological lesion. The nodular histological type of KS was the commonly identified histological type of KS and affected mostly males (40.5%, 34/84) than females (20.2 %, 17/84) and this was statistically significant (p=0.041) (Figure 3 B). The age group 21-40 years was the most affected by nodular lesions of KS compared to the other age groups. However, this difference was not statistically significant (p=0.199) (Figure 4).

**Figure 5:**
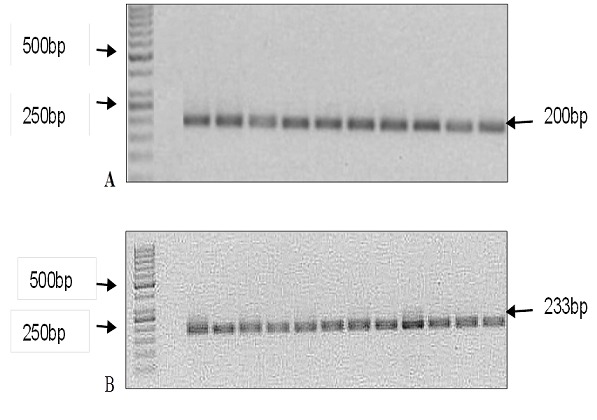
Detection of PCR products: A) human β-actin internal DNA extraction control-PCR detection of β-actin; Lane M: 50bp DNA marker, Lane 1, Negative control; Lanes 2-11 Representative KS tissue samples; B) PCR detection of HHV-8Lane M: 50 bp DNA marker, Lane 1: negative control, Lane 2: positive control, Lanes 3(RT058), 4(RT060),) 5(RT87), 6(RT161), 7(RT169), 8(RT173), 9(RT176) 10 (RT206) 11 (RT207): positive for HHV-8

**Figure 6:**
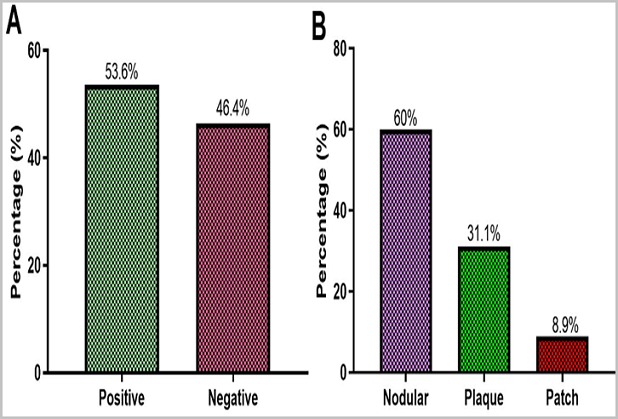
A) detection of HHV-8 in KS tissues; B) in various KS histological types

**Figure 7:**
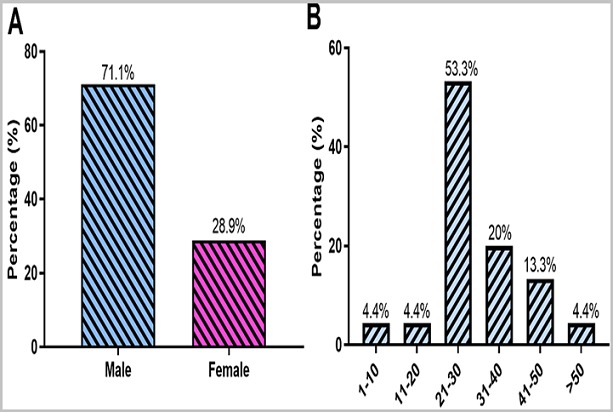
Detection of HHV-8 DNA based on A) gender; B) age

**Detection of HHV-8 ORF26 gene:** The presence of HHV-8 DNA was confirmed by running nested PCR products on a 1.5% agarose gel and a product size of 233bp was observed as shown in Figure 5. Out of the 84 confirmed KS tissues, HHV-8 DNA was detected in 53.6% (45/84) of the tissues (Figure 6 A). HHV-8 DNA was detectable in the tissues with the following histological distribution: Nodular (60%, 27/45), Plaque (31.1%, 14/45) and Patch (8.9%, 4/45). The nodular stage was more likely to harbour HHV-8 DNA than the other histological types of KS (OR=1.43, 95%CI 0.06 -2.04, p=0.035) (Figure 6 B). HHV-8 DNA was mostly detected in males (71.1%, 32/45) (p=0.493) than in females (28.9%, 13/45) (Figure 7 A) and mostly affecting the age group 21-30 years (53.3%, 24/45) (p=0.359) (Figure 7 B).

## Discussion

HHV-8 has been proven to be the causal agent of all forms of KS [20, 21]. The higher (93%) HHV-8 prevalence among KS cases compared to non-KS tumours and reactive lesions in a study conducted in Tanzania support a causal relationship between the virus and primary KS [14]. Detection of HHV-8 by means of PCR is important for making differential diagnosis of KS, and can be used for the confirmation of histopathological diagnosis especially in cases of early vascular lesions which are difficult to diagnose [22]. Epidemiological studies have indicated that HHV-8 seropositivity in various populations is strongly correlated with the population's risk of developing KS [22, 23]. In this study, three histological types of KS were identified: patch, plaque and nodular types. Patch is an early vascular lesion which mostly presents diagnostic challenges to the pathologists as it may be misdiagnosed because of its resemblance to other vascular lesions such as haemangiomas, haematomas and purpuras [24]. Plaque is the intermediate stage in which there is moderate proliferation of spindle-shaped cells while the nodular type is an advanced stage of the disease which, in most cases, poses no diagnostic challenges. Data in this study also demonstrated that the nodular type of KS was the commonly diagnosed histological type. These findings are consistent with those in a study conducted in Tanzania in which most of the KS cases (68.7%, 82/120) were identified at this stage [14]. The similarities in the two studies can be attributed to the fact that both countries are in the endemic region of KS where such results are expected. It could also be attributed to misdiagnosis at an early stage of the disease as the lesions are easily identified at an advanced stage or it may be that patients report to the hospital when the disease reaches an advanced stage [22].

This study also showed that the occurrence of KS was proportionally higher in men than in women. Men presented with more nodular lesions of KS compared to women. These findings are similar to studies carried out in other sub-Saharan countries [25, 26]. A Ugandan report showed that women were less likely to have nodular lesions of KS (OR=0.33, 95% CI 0.16-0.69 p=0.003) as compared to men [27]. The reduced rate of KS among females may be due to gender related factors that include hormonal, environmental or genetic factors which normally protect women against the disease. Human gonadotrophin has been hypothesised to be a protective factor in KS development based on its inhibition of the growth of KS cell lines in vitro and oestrogen has also been known to have direct effects on KS cell proliferation or by its direct effect on anti-tumour immune response [27]. Data presented in this study also demonstrates the presence of HHV-8 DNA in more than half of the KS tissues analysed. Tumour biopsies are a convenient source of viral DNA as they have a high viral load compared to peripheral blood [4]. A recent study employing nested PCR to evaluate the frequency of HHV-8 infection in HIV infected patients with and without KS manifestations in Brazil detected HHV-8 DNA in 100% (13/13) of the tissues analysed [22]. This was attributable to the pathogenic role of HHV-8 in KS. An Iranian study reported the presence of HHV-8 DNA in 83.3% (30/36) of the KS tissues [1]. Several other studies have also reported high detection rates of HHV-8 DNA in KS tissue biopsies [28–30]. Our findings along with those from other studies emphasize the pathogenic role of HHV-8 in the development of KS [31, 32]. The differences with this study in which a lower detection rate was obtained, may be attributed to formalin fixation in the tissues used. Formalin fixation results in widespread cross linkage between nucleic acids and proteins, with the result that DNA extracted from fixed tissues is fragmented into sequences of variable size, making it difficult to amplify the viral DNA [33]. It is noteworthy that several authors have observed the detectable presence of HHV-8 to be intermittent, perhaps contributing to the overall lack of sensitivity of PCR in detecting HHV-8 infection [34]. It has also been observed that the sensitivity of PCR depends on the accuracy and location of the excised biopsy. However, most biopsies are obtained in sites where there is less bleeding and this compromises the quality of the samples collected as the most aggressive lesion is not excised [24].

In this study, HHV-8 detection was higher in the nodular lesions of KS than other lesions. The nodular lesions were more likely to harbour HHV-8 DNA (OR=1.43 95% CI 0.06-2.04 p=0.035) than the other histological types of KS. These findings were consistent to those in a US study in which higher levels of HHV-8 DNA were also reported in the nodular stage compared to the patch and plaque stages [35]. Nodular lesions are the commonly diagnosed KS lesions, and semi-quantitative analysis has established that the nodular stage is associated with a higher viral load than the two other stages [35, 36]. Data obtained in this study strongly suggest a pathogenic role of HHV-8 in KS. Therefore HHV-8 detection has potential applications in the early diagnosis, staging and monitoring of KS lesions. This study also demonstrated that HHV-8 DNA detection was high in the 21-40 age group and in male patients, with no statistical significance with regard to age and sex. Similar results were obtained in a study conducted in Germany in which HHV-8 DNA was mainly detected in the middle-aged group (p>0.05) and was mostly seen in males (p>0.05) than in females [37]. High HHV-8 detection in the middle aged group in the two studies may be attributable to the high HIV risk due to high risk behaviour in this age group [25]. However, we could not attribute our findings to HIV infection due to lack of clinical data. The high detection rate in women may be attributed to gender- related factors such as hormonal, environmental and genetic factors which protect women against KS [27]. Studies conducted in Cameroon, China and Brazil have shown that HHV-8 prevalence increases significantly with age and is not related to sex [20, 38]. Local risk factors, geographical locations or ethnic makeup of the study population could also influence results obtained in the different studies [19, 39].

## Conclusion

Data generated in this study shows that KS lesions consist of three histological types: patch, plaque and nodular lesions. Of the three, the nodular lesion was the most common (60.7%) and was mainly identified in males than in females, supporting the notion that KS is a male- associated disease. Another significant finding was the detection of HHV-8 DNA in more than half of the KS tissues analysed, and this was mostly detected in the nodular lesions. Our data corroborate those from other studies suggest a role of HHV-8 in KS pathogenesis. We propose detection of HHV-8 in tissues by PCR as a way of making a definitive diagnosis for KS, especially in early vascular proliferations when the characteristic histopathological features are not present.

### What is known about this topic

- It is already known that HHV-8 is aetiologically linked to KS;
- HHV-8 infection is more common in males than females.

### What this study adds

- This study is the first of its kind to be done in Zambia and hence adds to the knowledge that HHV-8 is detectable in FFPE tissues of KS;
- The nodular KS are the most common type of KS in Zambia.

## Competing interests

The authors declare no competing interest.
